# Multi-frequency sono-fermentation with mono and co-cultures of LAB synergistically enhance mulberry juice: Evidence from metabolic, micromorphological, sensorial, and computational approaches

**DOI:** 10.1016/j.ultsonch.2024.107117

**Published:** 2024-10-22

**Authors:** Sanabil Yaqoob, Aysha Imtiaz, Ibrahim Khalifa, Sajid Maqsood, Riaz Ullah, Abdelaaty A. Shahat, Fahad Al-Asmari, Mian Shamas Murtaza, Jian-Ya Qian, Yongkun Ma

**Affiliations:** a School of Food and Biological Engineering, Jiangsu University, Zhenjiang, China; b Department of Food Science and Technology, Faculty of Science and Technology, University of Central Punjab, Lahore, Pakistan; c School of Food Science and Engineering, Yangzhou University, Yangzhou, China; d Food Technology Department, Faculty of Agriculture, Benha University, Moshtohor 13736, Egypt; e Department of Food Science, College of Agriculture and Veterinary Medicine, United Arab Emirates University, Al-Ain 15551, United Arab Emirates; f Department of Pharmacognosy, College of Pharmacy, King Saud University, Riyadh, Saudi Arabia; g Department of Food and Nutrition Sciences, College of Agriculture and Food Sciences, King Saud University, Saudi Arabia

**Keywords:** Multi frequency ultrasonication, Lactic acid bacteria, Co-culture, Metabolites, Interaction

## Abstract

The effect of multi-frequency ultrasound-assisted (20/28/40 KHz) lactic acid bacteria (LAB- *Lacticaseibacillus casei, Lactiplantibacillus plantarum, Lacticaseibacillus paracasei, Lactobacillus acidophilus,* and *Lactobacillus helveticus)* fermentation (mono and co-cultures) on the metabolic, structural, micromorphological, and sensorial properties of mulberry juice were evaluated. Results indicated that multi-frequency ultrasound-assisted fermentation significantly modified the microstructure of mulberry juice powder, resulting in more porous and rougher surfaces with irregular indentations. Total phenolic content in the best-performing sample (S10) increased to 365.36 mg GAE/mL, while total flavonoid content rose to 139.20 mg RE/mL (p < 0.05). Antioxidant activity, as measured by DPPH and FRAP assays, also showed considerable improvement, with DPPH scavenging activity increasing to 87.45 % and FRAP-value to 3.27 mM TE/mL (p < 0.05). Additionally, HPLC-UV analysis revealed that the amendment in the concentrations of cyanidin-3-rutinoside (47.47 mg/L) and peonidin-3-O-glucoside (66.86 mg/L) in the S2-based sample. E-nose analysis demonstrated intense flavor profiles in fermented samples, particularly in sample S15. Sensory evaluation also highlighted that the fruity and floral aromas in co-culture fermented samples were enhanced, notably in S10, S7, and S14. Thus, combining multifrequency ultrasonication and fermentation significantly enhances the antioxidants capacity, flavor profile, micro-morphology, and overall quality of mulberry juice.

## Highlights

• Mulberry juice was fermented by lactic acid bacteria with multi-frequency ultrasound.
• Mono and co-cultures bacteria were used.
• Metabolic, micromorphological, and sensorial properties of mulberry juice were measured.
• Sono-fermentation enhances the antioxidants capacity and flavor profile of mulberry juice.
• Sono-fermentation enhances the micro-morphology and overall quality of mulberry juice.

## Graphical abstract

**Figure:**
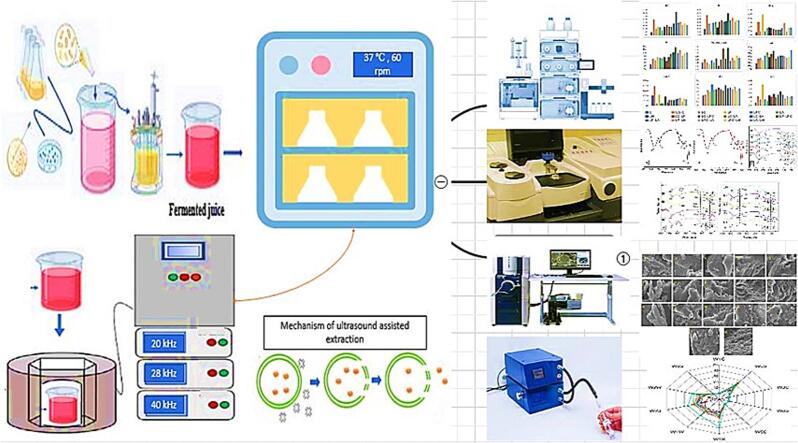

## 1 Introduction

Mulberry juice is a highly nutritious and functional beverage derived from the mulberry tree fruit, renowned for its richness in bioactive compounds including polyphenols, vitamins, and minerals [36]. However, the delicate and perishable nature of mulberry fruits presents significant challenges for their commercialization and preservation as fresh produce [17]. Traditional fermentation techniques have been explored to enhance the stability and functionality of fruit-based beverages [14]. However, the combination of multi-frequency ultrasound with lactic acid bacteria (LAB) fermentation represents an innovative approach to overcome these challenges.

The novelty of this study lies in the use of multi-frequency ultrasound (20/28/40 KHz) in conjunction with mono and co-cultures of LAB to enhance the metabolic, microstructural, and sensory properties of mulberry juice. While ultrasound and fermentation have each been studied independently for their effects on food products [31], [35] their synergistic application in this study uniquely contributes to improving the antioxidant capacity, flavor profile, and bioactive compound extraction from mulberry juice. This dual-treatment strategy has not been thoroughly explored in the literature, particularly regarding its impact on both the nutritional and sensory quality of fermented beverages. This study, therefore, presents a novel method for producing high-quality, functional mulberry juice with enhanced health benefits and consumer appeal [15], [45].

Explorative studies have identified numerous health-promoting and disease-ameliorating compounds in mulberry fruits, such as moranoline, albafuran, albanol, morusin, kuwanol, calystegine, and hydroxymoricin, which effectively regulate metabolic activities. Additionally, the distinctive flavor and appealing taste of mulberry-based beverages, enriched with anthocyanins and phenols, make them highly desirable to consumers [15].

Ultrasound technology, particularly multi-frequency ultrasound, has gained attention for its ability to enhance the fermentation process. Ultrasound works by generating acoustic cavitation, a phenomenon where the rapid formation and collapse of microscopic bubbles induce localized high temperatures and pressure gradients. This leads to the disruption of cell walls, increased mass transfer, and improved extraction of bioactive compounds [31]. When combined with fermentation, ultrasound can significantly improve the release of bioactive compounds, such as phenolics and flavonoids, by breaking down plant cell walls and enhancing enzyme activity. Ultrasound-assisted fermentation (UAF) in the genesis of functional foods is a conglomeration of techniques with in situ kindling-induced changes, intended to effect an ultraefficient and rapid fermentation [12]. Recently, ultrasound-assisted fermentation has gained attention as a novel approach to enhance the fermentation process. The use of multifrequency ultrasound in the mono and co-culture fermentation of mulberry juice has yielded promising improvements in the metabolic, structural, micromorphological, and sensory qualities of the final product [35]. The application of multifrequency ultrasound has been shown to significantly boost the extraction of bioactive compounds, such as polyphenols and vitamins, from mulberry fruits, thereby enhancing the nutritional profile of the juice. Moreover, ultrasound-assisted fermentation synergistically increases the production of health-promoting compounds, including moranoline, albafuran, and morusin, which play a pivotal role in regulating metabolic functions [43].

Considering the growing demand for nutrient-dense and high-quality foods, ultrasound-assisted fermentation, specifically utilizing multifrequency ultrasound, offers an innovative and efficient method for producing functional beverages like mulberry juice. This technique not only enhances the concentration of bioactive compounds within the juice but also effectively addresses commercialization and preservation challenges, thereby catering to consumer demands for health-promoting and sensory-pleasing products. This study aims to evaluate the effects of multi-frequency ultrasound-assisted fermentation on the phenolic content, antioxidant activity, and sensory qualities of mulberry juice. By combining ultrasound and fermentation, we hypothesize that this dual-treatment method will result in a significant enhancement of the bioactive properties and consumer acceptability of mulberry juice. Additionally, we aim to provide a comprehensive understanding of the underlying mechanisms involved in the interaction between ultrasound and LAB fermentation, including its impact on the microstructure, flavor profile, and bioavailability of key metabolites. The treatment codes for each sample were tabulated in Table S1.

## 2 Materials and methods

For this study, mulberry fruit (*Morus rubra*) were obtained from a farm in Zhenjiang, Jiangsu, China in May 2023. The fully ripened red mulberry fruit was manually picked, sorted, and washed for further use. The fruit was stored at −18 °C. Fruit was blended through laboratory blender. *Lacticaseibacillus casei* ATCC 393*, Lactiplantibacillus plantarum* ATCC 8014*, Lacticaseibacillus paracasei ATCC 334, Lactobacillus acidophilus* ATCC 4356, and *Lactobacillus helveticus* ATCC 15009, which were bought from SynBio Tech, Beijing, China, were propagated on MRS medium at 37 °C and stored at 4 °C. All other chemicals were of analytical grade.

### 2.1 Multifrequency ultrasonication-assisted fermentation conditions

The multifrequency ultrasonication-assisted fermentation of mulberry juice was carried out based on optimal conditions determined in previous experiments. For this process, lactic acid bacteria strains (*Lacticaseibacillus casei, Lactiplantibacillus plantarum, Lacticaseibacillus paracasei, Lactobacillus acidophilus, and Lactobacillus helveticus)* were cultivated in MRS broth in an incubator (HZQ-F160) to achieve a bacterial concentration of 10^7^–10^8^ CFU/mL. A 2 % bacterial suspension was then added to the mulberry juice, and the mixture was incubated at 37 °C with gentle shaking (60 rpm) for 4 h.

The ultrasound treatment was performed using a hexagonal ultrasonic bath developed by the School of Food & Biological Engineering at Jiangsu University (Zhenjiang, China). After 4 h of fermentation, 100 mL of the mulberry juice was transferred into a beaker and subjected to ultrasonication. The mulberry fruits (*M. rubra*) used in this study were sourced from a farm in Zhenjiang, Jiangsu, China (Zhenjiang Zhongnong Sericulture Technical Service Co.; Latitude: N 40.76″, Longitude: W 73.98′) in May 2023. Fully ripened red mulberry fruits were manually harvested, sorted, and washed before being stored at −18 °C. The fruits were then blended in a laboratory blender to produce juice with an approximate Brix value of 12.5 % and a pH of 6.9, measured using a refractometer and pH meter.

**Fig. 1:**
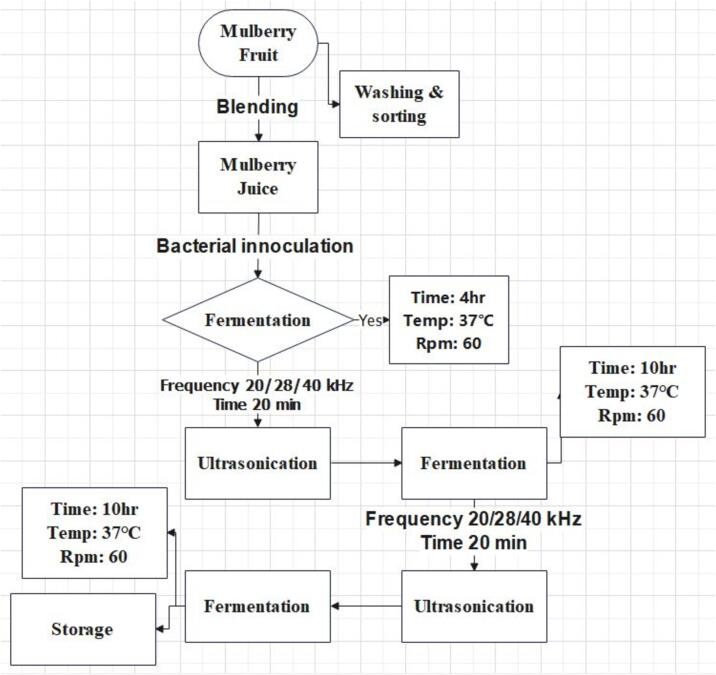
Flow chart of ultrasound assisted fermentation of mulberry juice with LAB.

For ultrasonication, a tri-frequency mode (20/28/40 kHz) was applied to each sample with an ultrasonic power density of 50 W/L. The pulse cycle was set to 10 s on and 10 s off, for a total treatment duration of 20 min [13]. The temperature of the ultrasonic bath was controlled at 25 ± 2°C using water recirculation. Following the ultrasonication treatment, the juice was incubated for an additional 10 h to complete the fermentation process. This cycle of ultrasonication followed by fermentation was repeated for a total fermentation time of 24 h (Fig. 1). The specific treatment conditions for each sample are listed in Table S1.

### 2.2 Extraction of phenolics

The extraction of free phenolic compounds from mulberry juice was performed [10]. Briefly, 1 mL of the mulberry juice was extracted with 20 mL of MeOH (80 %, v/v) and sonicated for 30 min. After centrifugation at 4000 g for 10 min, the supernatant was lyophilized and set as an undigested sample for further analysis.

#### 2.2.1 Total phenolic contents (TPC)

Phenolic contents of mulberry juice were find by the procedure of Mehmood et al. [25] with some modifications. Briefly, 1 mL Folin–Ciocalteu reagent, Na_2_CO_3_ 3 mL (20 % w/v), 12 mL H_2_O and 200 μL sample were mixed and placed in water bath (70 °C, 10 min). Finally, SpectraMax i3 spectrophotometer (Molecular Devices, Silicon Valley, CA, US) was used to check the absorbance at 765 nm. Gallic acid was used as a standard and results were expressed in mg of gallic acid equivalent/mL of sample.

#### 2.2.2 Total flavonoid content (TFC)

TFC of fermented mulberry juice were determined by the method of Chen, Chen, Xiao, and Fu [8] with modifications. Briefly, 25 μL sample, 0.1 mL H_2_O and NaNO_2_ 10 μL (5 %) were mixed and rested for 5 mins. After that AlCl_3_ 15 μL (10 %), NaOH 50 μL and H_2_O 50 μL were added. Finally, the absorbance was taken at 510 nm using SpectraMax i3 spectrophotometer. TFC was calculated using standard of quercetin and results were addressed in mg of rutin equivalent/mL of sample.

#### 2.2.3 Total flavonols content (TFlavC)

TFlavC of fermented mulberry juice were determined by the method of Kumaran and Karunakaran [22] with some modifications. Briefly, 2 mL AlCl_3_, 3 mL CH_3_COONa, and 2 mL sample was mixed and stored at 20 °C for 15 min. Finally, the absorbance was taken at 440 nm using SpectraMax i3 spectrophotometer. The TFlavCs were calculated using quercetin as a standard and results were expressed in mg of quercetin equivalent/mL of sample.

#### 2.2.4 Total anthocyanin contents (TAC)

TACs of fermented mulberry juice were determined by the method of Tchabo, Ma, Kwaw, Zhang, Li, and Afoakwah [41] through pH differential method with modifications. Briefly, two buffer solutions KCl (0.025 mol/l) and sodium acetate (0.4 mol/l) were prepared with pH 1 and pH 4.5, respectively. 200 μL was added in two sets of tubes followed by 1.8 mL of KCl buffer in one tube while 1.8 mL of sodium acetate buffer in other tube. The tubes were vortex, and the absorbance of each tube was measured at 700 and 520 nm using spectrophotometer (SpectraMax i3). The results were addressed as mg of cyanidin 3-glucoside equivalent/mL of sample. The calculation can be done through the following formula:

(1) TAC=[(K1-K2)-(C3-C4)]xMWxDFx100/εxL

Where K1 = Absorbance at 520 nm, pH1, K2 = pH1, Absorbance at 700 nm, C3 = pH4.5, Absorbance at 520 nm, C4 = pH 4.5, Absorbance at 700 nm, MW = Molecular weight of cyanidin 3 glucoside, DF = Dilution factor, Ɛ= molar extinction, L = path length.

#### 2.2.5 Proanthocyanidins contents determination (PACC)

PACC of fermented mulberry juice were determined by the method of Sun, Tsuang, Chen, Huang, Hang, and Lu [40] with some modifications. Briefly, 1 mL sample, 3 mL HCL and 6 mL vanillin were mixed and stored at 25 °C for 15 min. Finally, the absorbance was taken at 500 nm using SpectraMax i3 spectrophotometer. The PACC was calculated using standard of catechin and results were addressed in mg of catechin equivalent/mL of sample.

#### 2.2.6 Reducing power ability (Rp-A)

Rp-A of fermented mulberry juice were determined by the method of Natić et al. [29] with some modifications. Briefly, sample 1 mL, HCl 50 μL, C_6_N_6_FeK_3_ 400 μL, FeCl_3_ 400 μL and H_2_O 700 μL were mixed and dark incubated for 30 min at 37 °C. Finally, the absorbance was taken at 720 nm using spectrophotometer (SpectraMax i3). The results were addressed as mM of ascorbic acid.

#### 2.2.7 Copper reducing power ability (Cu-Rp)

Cu-Rp of fermented mulberry juice were determined by the method of Apak, Güçlü, Özyürek, and Karademir [3] with some modifications. Briefly, CuCl_2_ 250 μL (0.01 M), neocuproine 250 μL (7.5 mM), ammonium acetate 250 μL (1 M), and various concentration of sample were mixed. The solution was rested at RT for 30 min and the absorbance was measured at 450 nm.

#### 2.2.8 Determination of DPPH scavenging activity

The 2,2-diphenyl-1-picrylhydrazyl radical scavenging activity of the fermented juice was calculated using the method described by Ramirez, Zambrano, Sepúlveda, Kennelly, and Simirgiotis [37] with some modifications. First, DPPH (0.1 mM) was prepared in MeOH. 120 µL sample was mixed with 4.2 mL DPPH solution. After that the solution was rested for 30 min at RT and the absorbance was calculated at 517 nm. Fermented juice was used as control without DPPH solution and the scavenging activity were measured by following formula:

(2) DPPHscavengingactivity(%)=Control-sample/controlx100

#### 2.2.9 Determination of ferric reducing antioxidant power (FRAP)

FRAP of the fermented juice was evaluated [5] with some modifications. Briefly, FRAP solution was prepared by mixing acetate buffer 25 mL (0.3 M, pH 3.6), TPTZ 2.5 mL (10 mM, 40 mM HCl), and FeCl_3_·6H_2_O 2.5 mL (20 mM). 0.3 mL FRAP solution was mixed with 1 mL sample and 0.3 mL H_2_O. The mixture was stored at 37 °C in the water bath for 30 min and absorbance was measured at 595 nm. The results were calculated as mg of trolox equivalent/mL of sample.

### 2.3 Evaluation of phenolic compounds by HPLC-UV

The phenolic compounds were determined by utilizing the HPLC-UV Agilent 1260 infinity II system *via* adopting the methodology of Dou et al. [10], with minor modifications. The respective targeted analytes were eluted through C-18 column (Agilent Zorbax-SB; 4.6 mm 250 mm, 5 m particle size). The mobile phases mixture was as follows: Mobile phase A (0.1 % acetic acid) and mobile phase B (100 % acetonitrile). The linear gradient protocol was set as follows: 0–10 min, 5–10 % solvent B; 10–15 min, 10–20 % solvent B; 15–25 min, 20–38 % solvent B; 25–30 min, 38–40 % solvent B; 30–31 min, 40–100 % solvent B; 31–35 min, 100 % solvent B; 35–36 min, 100–5 % solvent B; 36–50 min, 5 % solvent B. The flow rate was 0.8 mL min^−1^, and the temperature of the column was m maintained at 30 °C. The chromatograms were recorded at 250, 284, and 520 nm phenolics, flavonoids, and anthocyanins, respectively. The phenolic compounds were qualitatively and quantitatively determined by comparing retention time and spectrum of standards with samples and quantified through peak area using calibration curves.

### 2.4 Micromorphology of samples

Scanning electron micrographs (PhenomTM) of each sample were obtained at magnifications of × 5000 [46]. Lyophilized samples were fixed on the holders with double spread and gold layer was sputtered on it and then scanned in a vacuum of 5kv potential difference.

### 2.5 Attenuated total reflectance-Fourier transforms infrared spectroscopy (ATR-FTIR)

FTIR spectra of all samples were obtained through ATR-FTIR (Thermo scientific, Nicolet iS50, Waltham, Massachusetts, US) thermo electron with attenuated total reflectance (ATR) accessory in the range of 4000–700 cm^−1^ [46].

### 2.6 Electronic nose analysis

Electronic nose analysis of fermented mulberry juice referred to a related method [47]. For each measurement, 10 mL of mulberry juice was placed in a 50 mL sample bottle, sealed, and analyzed using a PEN3 electronic nose (Airsense Ltd., Schwerin, Germany). Measurement conditions were of sensor cleaning time 150 s, sample preparation time 5 s, analysis time 120 s, internal flow rate 400 mL/min, and injection flow rate 200 mL/min.

### 2.7 Sensory analysis

The sensory evaluation of aroma characteristics of fermented mulberry juice was conducted by using quantitative descriptive analysis, referring to a previously described method [47]. 50 untrained food scientists aged 18 ∼ 30 years (25 males and 25 females) from the School of Food and Biological Engineering, Jiangsu University, Zhenjiang, China voluntarily evaluated the aroma characteristics of fermented mulberry juice. During the evaluation, the samples were randomly numbered and assigned. The five characteristic aroma attributes of fermented mulberry juice are floral (rose, violet, etc.), fruity (mulberry, banana, peach, etc.), mellow (alcohol), estery (cheese), and delicate (grass, green bean). The characteristic aroma evaluation grades of mulberry wine are 0 = no, 1 = very weak, 2 = weak, 3 = moderate, 4 = strong, and 5 = extraordinarily strong.

### 2.8 Molecular docking

The molecular docking technique was conducted *via* the educational version of MOE software, 2015, USA. The molecular interactions were performed between the structure of bacterial peptidoglycan (PDB ID: 2MTZ) as macromolecule receptor and cyanidin 3 rutinoside (C3R; PubChem CID: 4481459) as ligand which were retrieved protein data bank (https://www.rcsb.org/) and PubChem Database (https://pubchem.ncbi.nlm.nih.gov/). Both structures were then optimized by combining fractional charges, and energy was minimized using Protonate-3D and MMFF94X force fields, and H_2_O was removed, structure refining, energy minimization, and 3D protonation *via* MOE. The process was repeated for the composites, and thereafter 4–5 appropriate docked postures were created which were visualized and analyzed then for their hydrophobicity, electrostatics potential, H-bonds, and heat-map structure fluctuations *via* heatmapper (https://heatmapper.ca/expression/). Molecular dynamic simulation (MDs) runs were kept every 10 ns for a total of 50 ns, and the root mean square deviation (RMSD) was then expressed. Peptidoglycan-C3R geometry was first optimized *via* M062X operate with the 6-31G (d, p) aiding by Gauss 09 package. Alcalase was randomly located around peptidoglycan, double-minimized, and performed using GROMACS, ver., 5.1.4, GNU, Netherlands [21].

### 2.9 Data analysis

Data were processed using IBM SPSS Statistics 22 and origin 2022, principal component analysis (PCA) was conducted using SIMCA 14.1 by analyzing the three replicates done for each sample.

## 3 Results and discussion

### 3.1 Antioxidant activity

**Fig. 2:**
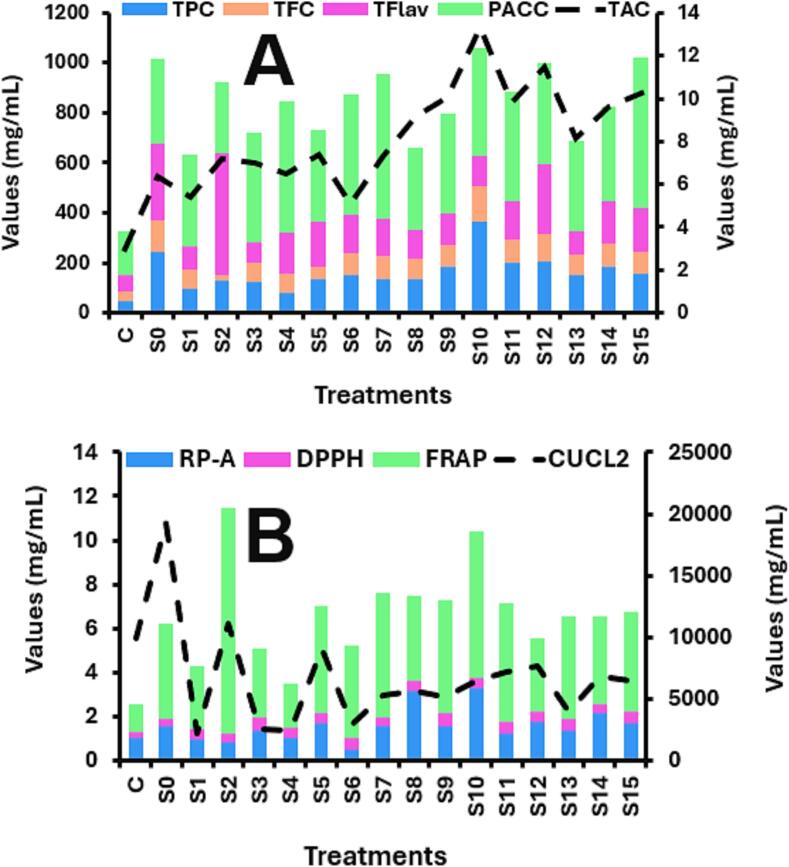
Effect of multi-frequency ultrasound assisted fermentation on the bioactive components **(A)** and their antioxidant effects **(B)**.

Fig. 2 illustrates the variation in basic antioxidant indexes of mulberry juice under the influence of multifrequency ultrasonication-assisted fermentation. This comprehensive assessment is necessary due to the **complex** nature of phytochemical contents and their reaction mechanisms, which cannot be adequately evaluated using a single method [11]. Bioactive compounds and *in vitro* antioxidant activities are crucial in determining the quality of juice. The results of TPC, TFC, TFlav, TAC, RP-A, PACC, CuCl_2_, FRAP, and DPPH radical scavenging activities are presented in Fig. 2.

There were significant differences in the bioactive compounds of mulberry juice. Compared to the control, TAC increases from 2.98 to 13.32 while all the fermented samples specifically S10 (13.32), S12 (11.44) and S15 (10.27) showed higher contents of anthocyanin. Anthocyanin is one of the main bioactive compounds in mulberry which can improve the astringency of fruit juice by binding with tannin [44]. TAC of monoculture fermented samples was significantly lower than that of the other groups (P < 0.05). Several factors may influence TAC, including the secondary metabolites released by LAB during the early stages of fermentation that react with anthocyanins, or the adsorption of monomeric anthocyanins in the juice by the cell walls of microorganisms [28]. Anthocyanins in mulberry juice are polymers of flavonoids, which can be decomposed into monomeric anthocyanins. TAC and other phenolic compounds play a key role in the antioxidant capacity of the juice [6].

The effect of multifrequency-assisted fermentation had a significant impact on TPC, TFC, TFlav, and PACC. Sample S10 exhibited the highest TPC (365.36) and TFC (139.20), followed by S0 with TPC and TFC values of 245.44 and 124.78, respectively. TFlav were highest in S2 (491.22), followed by S0 (307.67) and S12 (283.61). Proanthocyanidins were lower in the unfermented sample (175.75) and higher in S15 (600.50), S7 (580.75), S4 (526.75), and S6 (481.25). Meanwhile, the highest reducing power was noted in samples S10 (3.27) and S8 (3.17). In contrast, the CuCl_2_ assay values were higher in the S0 and control samples, with fermented samples showing a reduction in these values. Both DPPH and FRAP assays, conducted spectrophotometrically, indicated lower antioxidant activities in unfermented mulberry juice. However, the application of ultrasound-assisted fermentation markedly enhanced the antioxidant activity in these samples.

The formation of layers on the cell wall of the plant is the major barrier during the extraction process. Fermentation and ultrasonication are the best processing techniques to breakdown the layer under the influence of microorganisms and cavitation. Moreover, the bioactive compounds extraction also increases by ultrasonication and fermentation which may be due to conversion of macromolecules into their building blocks and high shear forces generated by acoustic cavitation [45]. Flavonoids may interact with anthocyanins to form more stabilized pyrano-furotheophylls, and the decrease in polarity of these compounds is accompanied by a decrease insolubility. Many scientists reported the positive effect of ultrasound treatment on antioxidant profile as it improves the bioavailability of bioactive compounds [25]. A previous study reported a significant increase in bioactive compounds with high antioxidant value due to the impact of ultrasonication [49]. Oxidative stress is the major cause of many diseases (cardiovascular, diabetes, and neurodegenerative disorders) and due to excessive reactive oxygen species production, the antioxidant system disturbs its activity and causes oxidative stress (1–4). Pan, Li, Lao, Hou, Gao, and Wu [33] reported a higher amount of FRAP and DPPH of pomegranate peel after ultrasound treatment. Pomegranate treated with fermentation and ultrasound technique showed higher polyphenolics and antioxidant activities [14].

### 3.2 Effect of LAB (mono and co-culture) on phenolics profile of mulberry juice

Phytochemicals are naturally occurring compounds found in plants that have been associated with a wide range of health benefits [23]. HPLC-UV was utilized to analyze the polyphenolic metabolites in mulberry juice produced through multi-frequency ultrasound-assisted fermentation, involving both mono and co-cultures of various lactic acid bacteria. Table 1 provides a comparative analysis of the phenolic components in fermented and non-fermented mulberry juice. The data were evaluated by measuring the peak areas at specific retention times and comparing them to the standards in the spectra of the different samples. The phytochemical profile of the final product can be influenced by the processing method used. Fermentation is one such technique that can impact the levels of polyphenols present in mulberry juice. According to Sharma, Garg, Kumar, Bhatia, and Kulshrestha [38], certain microorganisms like LAB can produce enzymes that hydrolyze conjugated polyphenols during the fermentation process. This increases the polyphenols' bioavailability and antioxidant capacity. Furthermore, specific fermentation conditions may promote the formation of extra phenolic compounds, hence increasing the health-promoting properties of products that are fermented. The increase in phytochemical concentrations can be attributed to the breakdown of plant cell walls and the subsequent release of cell-bound substances during fermentation by the enzymes of LAB. This process facilitates the release and utilization of these compounds, enhancing their availability in the final product.

**Table 1:** Phenolic profile of fermented mulberry juice identified using HPLC-UV.

**Sr. No**	**Compounds**	**MF**	**RT (min)**	**C**	**S0**	**S1**	**S2**	**S3**	**S4**	**S5**	**S6**	**S7**	**S8**	**S9**	**S10**	**S11**	**S12**	**S13**	**S14**	**S15**	**Biological activity**	**Reference**
**Anthocyanins**																						
01	Peonidin 3-o-glucoside	C_22_H_23_O_11_	16.2	12.22	14.68	32.65	66.86	34.00	34.42	51.63	45.90	41.26	33.18	30.32	41.80	44.29	36.66	43.50	41.7	28.87	Anti-inflammatory	Sari et al., 2019
02	Peonidin 3–5-glucoside	C_27_H_31_O_15_	15.8	7.87	9.40	8.72	16.13	7.68	11.86	8.17	8.39	7.63	7.76	9.24	11.68	8.46	9.02	7.67	7.73	8.61	Cardio protective agent	Krga et al., 2019
03	Cyanidin-3-rutinoside	C_27_H_31_O_15_	14.98	34.25	44.35	44.45	45.36	44.46	44.27	44.60	44.58	44.19	44.45	44.71	47.47	44.61	44.31	44.44	44.5	44.22	Neuro protective agent	Liang et al., 2021
**Total**				**54.34**	**68.43**	**85.83**	**128.34**	**86.14**	**90.55**	**104.4**	**98.87**	**93.09**	**85.39**	**84.27**	**100.95**	**97.35**	**89.99**	**95.61**	**94.02**	**81.70**		
**Flavoniods**																						
04	Morin	C_15_H_10_O_7_	27.1	0.47	2.87	2.88	2.99	2.90	2.98	2.92	3.72	3.75	3.79	2.94	3.76	2.95	3.64	3.04	3.38	3.11	Antioxidant, anti-inflammatory	Kuzu et al., 2019
05	Rutin	C_27_H_30_O_16_	20.01	25.42	31.12	31.50	41.09	31.88	32.27	35.41	35.28	33.83	31.59	30.44	34.25	34.17	32.22	31.92	34.45	31.27	Anti plasmodic, hypolipidemic	Patel and Patel, 2019
06	Quercetin	C_15_H_10_O_7_	27.6	2.31	3.01	3.01	2.84	3.01	2.95	2.99	3.96	3.95	3.95	3.03	3.96	3.00	3.99	4.12	3.07	3.16	Anti immunosuppressor	Yang et al., 2020
07	Catechin	C_15_H_14_O_6_	15.8	21.61	27.62	29.19	28.99	26.73	24.04	23.41	28.97	28.60	23.79	23.39	25.60	25.61	23.32	25.36	24.50	25.12	Viral infection prevention	Juca et al., 2020
**Total**				**49.80**	**64.62**	**66.58**	**75.90**	**64.52**	**62.23**	**64.74**	**71.93**	**70.13**	**63.13**	**59.80**	**67.58**	**65.72**	**63.17**	**64.44**	**65.4**	**62.66**		
**Phenolics**																						
08	Quinic acid	C_7_H_12_O_6_	34.00	76.18	174.47	127.94	173.77	85.13	100.10	126.73	87.74	82.76	73.27	83.52	133.37	79.35	76.18	201.01	243.4	182.31	Anti-cancer, antidiabetic	Ahmed et al., 2023
09	Coumaric acid	C_9_H_8_O_3_	20.20	0.12	3.34	1.62	17.70	2.93	3.62	10.44	8.49	6.01	2.63	1.45	10.07	7.96	5.19	22.15	7.41	15.33	Anti-melanogenic	Boo et al., 2019
10	Gallic acid	C_7_H_6_OH_5_	3.11	26.76	28.31	29.40	29.87	29.57	29.57	29.61	30.73	31.68	30.91	28.71	31.57	29.06	28.79	29.44	31.15	31.55	Antidiabetic, anti-microbial	Zahrani *et al.,* 2020
11	Chlorogenic acid	C_16_H_18_O_9_	14.87	6.13	7.76	6.21	7.74	6.36	6.46	7.12	6.97	6.59	6.33	6.31	6.88	6.85	6.37	5.90	6.63	5.87	Anti-carcinogenic	Lu et al., 2020
12	p-hydroxy benzoic acid	C_7_H_6_O_3_	15.03	6.75	8.85	7.91	8.00	7.90	7.94	8.19	8.23	8.26	8.21	8.10	8.21	8.37	8.06	9.80	8.35	9.28	Anti-bacterial	Wang and jiang,2022
13	Ferulic acid	C_10_H_10_O_4_	21.50	2.03	1.96	2.42	2.92	2.10	2.28	2.75	2.72	2.65	2.41	2.32	2.55	2.78	2.48	3.49	2.57	4.08	Vascular disorder prevention	Li et al., 2021
14	Cinnamic acid	C_9_H_8_O_2_	27.20	5.35	7.27	7.24	8.40	6.57	6.83	7.30	6.89	6.79	6.63	6.59	6.78	6.67	6.80	7.80	8.71	7.47	Cancer and neurological	Ruwizhi and aderibigbe, 2020
15	Procatenoic acid	C_7_H_6_O_4_	28.40	2.48	2.30	2.27	3.10	3.19	3.20	3.24	3.41	3.37	3.27	2.34	3.28	3.06	3.45	2.06	2.60	2.06	Analgesic effect	Benali et al., 2022
16	Vanilic acid	C_8_H_8_O_4_	16.20	7.37	9.52	10.98	10.81	11.87	11.54	11.58	11.86	11.64	11.75	11.67	23.70	11.12	13.04	9.63	9.86	10.62	Recover hyperlipidmia	Shen et al., 2019
17	Sinapic acid	C_11_H_12_O_5_	21.19	5.33	6.64	5.85	12.33	7.24	8.24	8.09	6.90	6.46	5.86	5.74	7.20	7.19	6.30	6.91	9.11	6.76	Hepatoprotective agent	Yan et al., 2020
18	Neochlorogenic acid	C_16_H_18_O_9_	10.13	27.02	30.71	30.85	42.83	33.21	32.04	37.67	35.86	34.89	31.99	33.62	34.50	32.04	34.24	34.34	35.39	33.44	Alleviate toxicity	Silva et al., 2021
19	Syringic acid	C_9_H_10_O_5_	17.20	6.60	8.88	14.65	16.52	13.13	12.94	10.59	10.28	10.20	12.86	12.84	26.54	15.21	16.66	6.61	27.46	7.33	Anti-bacterial effect	Wang and Jiang, 2022
20	Tri hydroxy benzoic acid	C6H2 (OH)3CO2H	8.20	44.05	44.21	44.28	45.78	44.25	44.25	44.40	44.45	44.41	44.31	44.24	44.40	44.43	44.27	44.47	44.22	44.31		
**Total**				**216.1**	**334.2**	**291.6**	**379.7**	**253.4**	**269.0**	**307.7**	**264.5**	**255.7**	**240.4**	**247.4**	**339.0**	**254.0**	**251.8**	**383.6**	**436.8**	**360.4**		
**Total of polyphenols**				**320.3**	**467.2**	**444.0**	**584.0**	**404.1**	**421.8**	**476.8**	**435.3**	**418.9**	**388.9**	**391.5**	**507.5**	**417.1**	**404.9**	**543.6**	**596.2**	**504.7**		

**1.** C-Control; **2.** LC-Lacticaseibacillus casei; **3**. LP-Lactiplantibacillus plantarum; **4.** LPC-Lacticaseibacillus paracasei; **5.** LA-Lactobacillus acidophilus; **6.** LH-Lactobacillus helviticus; **7.** LC-LP Lacticaseibacillus casei-Lactiplantibacillus plantarum; **8**. LC-LPC Lacticaseibacillus casei- Lacticaseibacillus paracasei; **9.** LC-LA Lacticaseibacillus casei- Lactobacillus acidophilus; **10**. LC-LH Lacticaseibacillus casei- Lactobacillus helviticus; **11**. LP-LPC Lactiplantibacillus plantarum- Lacticaseibacillus paracasei; **12**. LP-LA Lactiplantibacillus plantarum-Lactobacillus acidophilus;**13.** LP-LH Lactiplantibacillus plantarum-Lactobacillus helviticus; **14.** LPC-LA Lacticaseibacillus paracasei- Lactobacillus acidophilus;**15**. LPC-LH Lacticaseibacillus paracasei - Lactobacillus helviticus;**16.** LA-LH Lactobacillus acidophilus - Lactobacillus helviticus.

According to Ojha et al. [31], ultrasound-assisted fermentation has recently emerged as a promising strategy for increasing the amount of bioactive compounds in different foods. During the fermentation process, numerous compounds are converted into new substances and the bioavailability or number of components increased. Phenolics synthesis begins with glucose, which serves as a precursor. In the following phase, glucose enters routes such as the phenyl propane pathway and glycolysis, which are responsible for the conversion and novel synthesis of phenolic substances [19]. The process of fermentation in which important chemicals in raw materials proliferate and break down, resulting in the production of novel substances. At the exact same time, additional chemicals produced during fermentation are released, resulting in the production of bound phenolics. Bound phenolics contribute in a dynamic procedure in which they bound and released at an alternate rate than native phenolics, ultimately increasing the phenolic amounts of the product through the process of fermentation [42]. According to Ismail, Yusuf, Pu, Zhao, Guo, & Liu, [18], phytochemicals linked to their polysaccharide subunits are less freely released. On the other hand, with ultrasonication, the chemical bond breakdown action boosts phenolic acid bond dissociation into their aglycon derivatives, releasing the phenolic component into the samples, as reported; accordingly, ultrasound-assisted fermentation might lead to a larger release of bound polyphenols [26]. A previous study has shown that adding fermentation to ultrasound treatment greatly enhances TPC and antioxidant capacity of several food products such as soybean and grape pomace [32]. The structure and composition of phenol compounds are altered by the enzymatic breakdown process, increasing the concentrations of kaempferol, *trans*-ferulic acid, quercetin and *trans*-p-coumaric acid in sprouted quinoa.

Regarding anthocyanins, higher values were observed, with the predominant compounds being peonidin-3-O-glucoside and cyanidin-3-rutinoside. The use of mono and co-cultures positively impacted anthocyanin levels, resulting in increases across most treatments. Cyanidin-3-rutinoside was highest in sample S10 (47.47) and lowest in the control sample (C) (34.25). Peonidin-3-O-glucoside was highest in samples S2 (66.86), S5 (51.63), S6 (45.90), S11 (44.29), S10 (41.80), and S14 (41.78), while it was lowest in the control sample (C) (12.22), S0 (14.68), and S15 (28.87). Peonidin-3–5-glucoside was found in the lowest amounts overall, with the highest amounts present in S4 (11.86) and S10 (11.68). Flavonoid content increased from 49.80 to 75.90 (Table 1). Rutin was the most abundant flavonoid, ranging from 25.42 to 41.09, followed by catechin (21.61–28.99), quercetin (2.31–4.12), and morin (0.47–3.79). Phenolic acids also showed a positive impact, with most phenolics increasing after fermentation. The concentrations of specific phenolic acids were as follows: Quinic (76.18–243.42), coumaric (0.12–22.15), gallic (26.76–31.68), chlorogenic (5.90–7.74), p-hydroxybenzoic (6.75–9.80), ferulic (1.96–4.08), cinnamic (5.35–8.71), procatechuic (20.6–3.41), vanillic (7.37–13.04), sinapic (5.33–12.33), neochlorogenic (27.02–42.83), syringic (6.60–27.46), and trihydroxybenzoic acids (44.05–45.78).

The inclusion of LAB during fermentation increased anthocyanin level compared to unfermented juice. This enhancement in quantity is attributed to the enzymatic breakdown capabilities of mono and co-cultured LAB. These bacteria produce specific hydrolytic enzymes that contribute to the stability of anthocyanins in fermented mulberry juice. Anthocyanins are particularly sensitive and unstable compounds, affected by changes in pH and reactions such as acylation or methylation, which occur at the fundamental structure of the anthocyanin, specifically at the A or B ring of the OH-group [2]. LAB's complex metabolic conversion pathways are clarified further by their potential for breaking down phenolic acids *via* reductase or decarboxylase activities, which are strain-specific [33]. Phenolic acid decarboxylase is an enzyme that breaks down phenolic acids into flavonoids, which is whether guaiacol and 4-vinyl guaiacol are produced. The efficiency of the internal microbial synergy of LAB in enhancing these bioactive compounds in the fermented mulberry juice is correlated with the evolution of phenolic substances. In summary, these results highlight the bioconversion of phenolic substances and strain-specific and enzymatic changes that occur during the co-culture fermentation of fruit juice by LAB.

### 3.3 Effect of ultrasonication assisted fermentation on micromorphological integrity *via* SEM

**Fig. 3:**
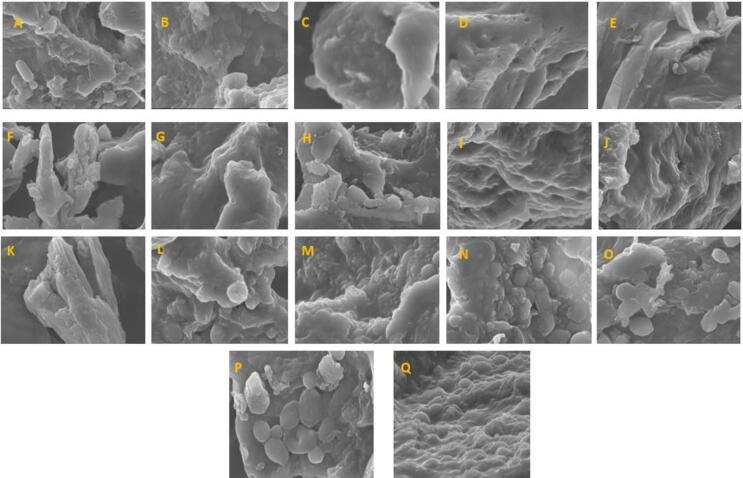
Scanning electron pictographs of different samples. **A.** C (Control), **B**. S_0_(US-C), **C.** S_1_ (LC), **D.** S_2_ (LP), **E.** S_3_ (LPC), **F**. S_4_ (LA), **G**. S_5_ (LH), **H.** S_6_ (LC-LP), **I**. S_7_ (LC-LPC), **J.** S_8_**(**LC-LA), **K**. S_9_ (LC-LH), **L.** S_10_**(**LP-LPC), **M.** S_11_ (LP-LA), **N**. S_12_ (LP-LH), **O.** S_13_ (LPC-LA), **P.** S_14_ (LPC-LH), and **Q.** S_15_ (LA-LH).

Ultrasonication and fermentation are considered pivotal processing techniques that significantly alter the overall properties of the product and completely transform the microstructure of food commodities. Ultrasound treatment enhances the solubility and emulsifying properties of food by modifying protein structures, leading to improved functionality and stability of the final product. Previous studies also elucidating its efficiency to improve texture, shelf life and digestibility of the food product by breaking complex carbohydrates into their building blocks [7]. These attributes of ultrasonication and fermentation are identical because both underwent the same characteristics. SEM was employed to identify the morphological characteristics of the samples. A comparison of SEM images between the control, ultrasonicated, and ultrasound-assisted fermentation samples revealed that the processed samples were more porous compared to the control. Specifically, the ultrasound-assisted fermentation sample exhibited a more porous and rougher surface (Fig. 3). The compact structure observed in the unfermented mulberry juice is due to the complex nature of its constituents. Ultrasonication induces irregularities and bond cleavage among various components, allowing compounds to move freely and become more accessible to LAB. Fermentation further disrupts granules and creates more indentations, leading to additional bond cleavage and increased interaction between compounds and bacteria. The micrograph of the unfermented sample revealed a smooth surface with small indentations, no visible fractures, robust structure and tiny pores [50]. The structure also showed visible continuous sheet-like structures that indicate the presence of macronutrients like protein, carbohydrates similar to the observations made by Zafar, Aldughpassi, Al-Mussallam, & Al-Othman, [48]. The surface of ultrasound assisted fermented samples with mono and co-culture bacteria displayed more grooves, irregular particles, and shallow indentations. It may be due to phase transformation and hydrolyzation that lead to suppressing and crumbling of granules and acquisition of irregular granules. This finding is consistent with the results of Wang et al. [43] who found that ultrasound reduces the size of the particles. This can be potentially attributed to the cellular deterioration effect generated when a combination of enzyme and ultrasound treatment methods was used [16].

Oh, Choi, Lee, Kim, and Moon [30] reported irregular, cracked, and disrupted granules in modified corn starch compared to native starch. A visible impact of ultrasound treatment can be noted in Fig. 3 as it degrades the cell wall of the sample which results in more irregular, rough, loosened and surface with sharp indentations. Ultrasound assisted fermentation through mono culture showed more smaller and irregular particles which lead to lower the water retention capacity, ultimately increased the water absorbing capacity, that eventually results in more compact bonding and better rheological properties [39]. The particles of ultrasound assisted fermented samples through co-culture showed heterogenous rough surface, more irregular indentations and large pores as depicted by [50]. They elaborated the impact of LAB causes degradation of macro-nutrients, which release the availability of phenolics, amino acids, organic acids, and other bioactive and nutraceutical compounds from the juice through improving the digestibility and metabolic efficiency of the product. These alterations indicate that fermentation, ultrasonication and ultrasound assisted fermentation improve the nutritional profile, texture, flavor, and shelf life of juice. The results concluded the impact of cavitation, resulting in sponge effect, which causes loosened structures and indentations on surface. This could also increase the quantity of metabolites and its bioavailability because most of the connections among constituents are broken, resulting in increasing the formation of lactic acid. The breakage of bonds due to double effect through ultrasound and fermentation treatment increased the surface area, improve bioavailability and flavor of the juice [1].

### 3.4 Influence of ultrasonication assisted fermentation on structural integrity *via* FTIR

**Fig. 4:**
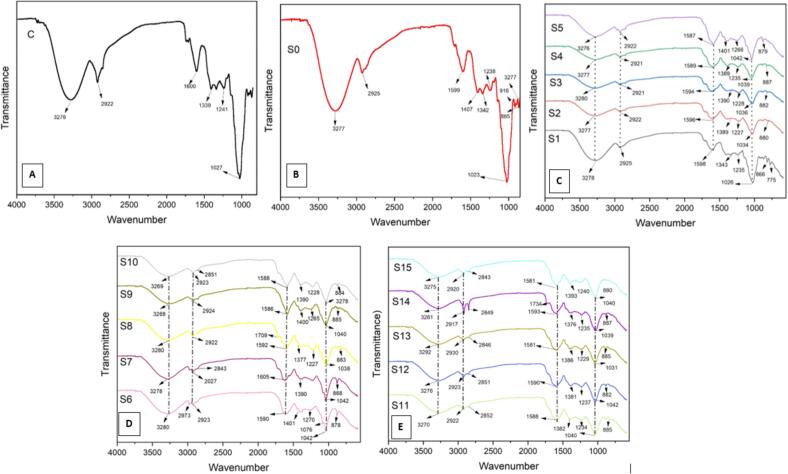
FTIR peaks of different samples. **A** is for the control samples (C). **B** is for US-C (S_0_), **C** is for LC (S_1_), LP (S_2_), LPC (S_3_), LA (S_4_), and LH (S_5_) samples. **D** is for LC-LP (S_6_), LC-LPC **(**S_7_), LC-LA **(**S_8_), LC-LH **(**S_9_), and LP-LPC **(**S_10_) samples. **E** is for LP-LA (S_11_), LP-LH (S_12_), LPC-LA (S_13_), LPC-LH (S_14_), and LA-LH (S_15_) samples.

FTIR-spectroscopy is one of the most effective techniques for understanding molecular interactions, identifying functional groups, confirming the presence of various molecules, and observing changes in samples due to fermentation. The FTIR spectra (4000–700 cm^−1^) of the multi-frequency ultrasound-assisted fermented samples were analyzed (Fig. 4). Differences in band positions and intensities were observed after incorporating the bacteria. The mulberry juice exhibited distinctive peaks at 3278, 2921, 1339, 1240, and 1027 cm^−1^, indicating specific molecular interactions and functional groups present in the samples. The broad peak at 3278 cm^−1^ indicates strong stretching vibrations of OH functional groups, characteristic of H_2_O, reflecting the high-H_2_O content in fresh juice. The peak at 2921 cm^−1^ is associated with the presence of C–H and OH bending/stretching, indicating aromatic alcohols and alkanes. The peak at 1339 cm^−1^ signifies moderate OH activity of phenols and alcohols. The strong peaks at 1241 and 1027 cm^−1^ correspond to C—O and C—N stretching, suggesting the presence of amines and ethers.

The spectra of mono and co-culture fermented juice displayed several novel peaks, indicating that microbial incorporation altered the functional profile of the fruit juice due to fermentation. The main peaks were prominent at 3270, 2920, 2850, 1734, 1709, 1596, 1400, 1376, 1230, 1266, 1038, 916, 886, and 775 cm^−1^ for all the samples, with minor differences. The weak shoulders at 916, 866, and 775 cm^−1^ can be attributed to strong C–H and OH bending, related to the anomeric configuration of carbohydrates, and are more prominent in ultrasound-assisted co-culture fermented mulberry juice. The presence of organic acids confirmed by peaks in 1709 and 1734 cm^−1^ represents the C = O bond of carboxylic acids and C–H bending of aromatic compounds. These are regarded as novel peaks due to bacterial inoculation, potentially indicating the stretching vibrations of alkene groups, carboxylic acids, aldehydes, and carbonyl groups. The absorbance at 1408 cm^−1^ is attributed to ether linkages. The strong shoulder at 3270 cm^−1^ indicates O–H stretching due to alcohols and carboxylic acids and indicates the reduction of H_2_O and its conversion into other compounds. The shift of the peak from 3278 to 3270 cm^−1^ after bacterial fermentation represents the interaction to generate intermolecular hydrogen bonding. The peaks at 2920 cm^−1^ represent gallic acid, while the shift from 1339 and 1376 to 1408 cm^−1^ indicates an increase in alcohols and phenols, referencing the presence of OH functional groups. Additional peaks at 866, 880, 886, and 817 cm^−1^, observed at the beginning of the spectra, result from bacterial action during fermentation, showing the vibration of CH_2_. Absorbances related to C—O bond stretching correspond to the fingerprint region of the spectra.

Fermentation with probiotics produces aromatic amine groups, indicating the presence of bacterial proteins in the final fermented product, which have antibacterial properties in different fermented samples. A study by Panda, Sahu, Behera, and Ray [34] found various phenol groups, aliphatic amines, alcohols, and carboxylic acids at 1000, 1250, and 3270 cm^−1^ peaks in fruit and purple sweet potato extracts. During fermentation, bond rearrangements occur, such as C–H stretching vibration changes from 2928 to 2925 cm^−1^, indicating H-bond formation. [24] revealed that H-bonding at the glycosidic bond between O_2_ and the presence of OH-group at the 886 cm^−1^ absorption peak. The amide I peak (1600–1700 cm^−1^) was primarily associated with C=O stretching vibrations, while amide II (1500–1600 cm^−1^) and amide III (1200–1400 cm^−1^) peaks were primarily associated with N—H bending vibrations and C—N stretching vibrations, respectively. The peaks corresponding to the amide region shifted when the samples were subjected to ultrasound and fermentation treatment methods. The amide III peaks shifted from 1241 (C) to 1227 cm^−1^ and 1339 to 1401 cm-^1^. These changes can be potentially attributed to the reduction in the number of hydrogen and internal disulfide bonds in the systems. This decrease in the number of bonds can be attributed to the internal breakage of molecules under conditions of combined effect of ultrasonication and fermentation [9]. This eventually results in changes in the secondary structure of juice.

Many positive chemical changes occur during fermentation, improving intermolecular crosslinking and sometimes altering molecular structure to create new covalent bonds, protein interactions, and bioactive compounds [20]. Fermentation changes H-bonding and increases intermolecular covalent cross-linking, enhancing juice quality. FTIR results are supported by Panda et al. [34], who fermented sapote fruit to produce wine, identifying peaks at 1201, 1459, 2847, 2925, and 3025 cm^−1^ linked to secondary amides, aromatic esters, O–H stretching compounds, and C–H stretching. These differences occur between 700–2000 cm^−1^, linked to fermentation and the formation of new compounds like phenols, organic acids, glycerol, and alcohol. Peaks at 1352 and 1644 cm^−1^ indicated amides II and I, linked to interactions between amino and OH-groups that modified the secondary structure of pullulan film. The peak at 1148 cm^−1^ represented a typical saccharide structure [20].

### 3.5 E-Nose analysis

**Fig. 5:**
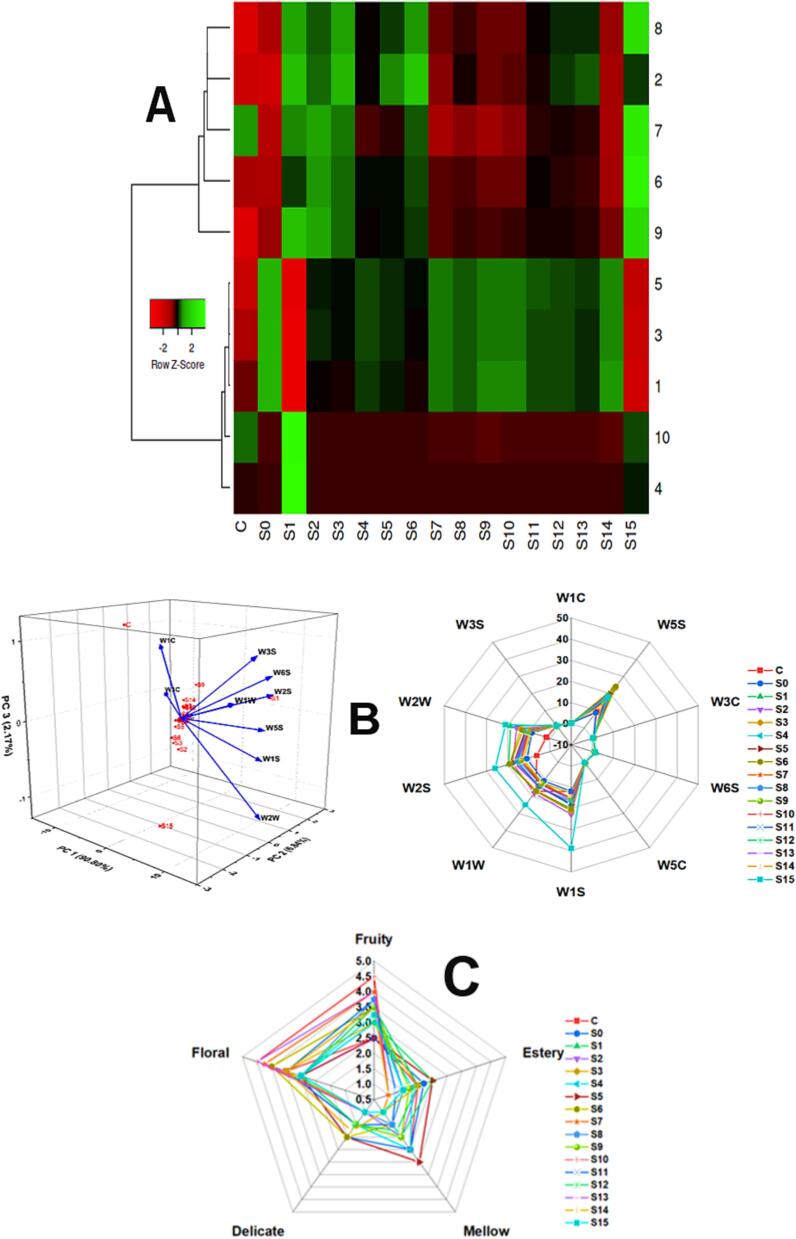
E-Nose analysis of different samples **(A)**. Samples codes as mentioned in Table 1. The sensors numbers from 1 to 10 are for W1C, W5S, W3C, W6S, W5C, W1S, W1W, W2S, W2W, and W3S which are sensitive for aromatic organic compounds, very sensitive, broad range sensitivity, reacts to nitrogen oxides, very sensitive with a negative signal, ammonia, also used as a sensor for aromatic compounds, detects mainly hydrogen gas, Alkanes, aromatic compounds, and nonpolar organic compounds, sensitive to methane, a broad range of organic compounds detected, detects inorganic sulfur compounds, for example, H2S, sensitive to many terpenes and sulfur-containing organic compounds, detects alcohol, partially sensitive to aromatic compounds, broad range, aromatic compounds, inorganic sulfur and organic compounds, and reacts to high concentrations (>100 mg kg^−1^) of methane and aliphatic organic compounds, respectively. PCA to distinguish flavor profile of samples and Radar plot of all samples **(B)**. Radar plot showing consumer acceptability via sensory evaluation **(C)**.

Flavor is a crucial quality attribute of mulberry juice. To distinguish the aroma profiles of juices incubated with different LAB strains and co-cultures, E-nose analysis was conducted. The maximum response values from 10 sensors of an electronic nose device are presented in radar plots (Fig. 5**A**). High responses were observed in the sensors W5S, W1S, W2S, W2W, and W1W, which represent high sensitivity to nitrogen oxides, methane, alcohol, and inorganic sulfides, respectively. These compounds are primarily responsible for the flavor development in the final product. The flavor profile of fermented samples was found to be superior to that of the control. Sample S15 exhibited the highest values. While both ultrasound and fermentation improved the flavor profile compared to the fresh material, the increase in elements such as nitrogen and sulfides may lead to undesirable odors in the products [45]. Tri-frequency ultrasound-assisted ethanol pretreatment also improved the flavor of scallion cylinders more than the pretreatment in dH_2_O [51].

Fig. 5**A** shows the mean values of the responses from different sensors, revealing distinct patterns across samples. Sample S0 exhibited higher levels of aromatic compounds (W1C), ammonia (W3C), and alkanes (W5C). Sample S6 had higher nitrogen oxides (W2S), while Sample S1 detected hydrogen gas (W6S) and methane (W3S). Sample S15 showed higher levels of organic compounds, was sensitive to terpenes, and detected alcohol and aromatic compounds. During fermentation, these compounds convert into flavor compounds, which is why all the treated samples had better flavor than the control. It can be concluded that multifrequency ultrasound-assisted fermentation impacts the flavor of the final products. A statistical test is needed to elucidate the changes in aroma under various ultrasound frequency modes.

Principal component analysis (PCA) is used to discriminate the difference among all samples (Fig. 5**B**). The sum PC_1_, PC_2_, and PC_3_ > 95 % and thus reveal that PCA analysis was viable for this dataset. PCA is a classical supervised data dimension reduction method that can be utilized to identify perceptible olfactory differences among various sources, narrowing differences among similar sources and widening differences among distinct sources, with more considerable distances among groups indicating more significant variability. Clustered different samples with good differentiation according to the fermenting bacteria and co-culture. Multi-frequency ultrasound assisted fermentation increased the concentration of aromatic compounds compared to control. The hypothesis is that a higher combination of frequencies combined with fermentation resulted in improved extraction of flavor compounds and limited degradation of flavor.

### 3.6 Sensory analysis for consumer acceptability

Sensory analysis of ultrasound-assisted fermented mulberry juice with different bacteria (mono and co-culture) revealed that the main flavor characteristics of the juice were fruity, floral, mellow, estery, and delicate. The intensity of each descriptive characteristic was quantitatively described, and the results are shown in Fig. 5**C**. In the fruity aroma profile, samples S10, S7, and S14 scored significantly higher than the other groups (P < 0.05). For the floral aroma profile, co-cultures, specifically S10, S7, S14, S12, and S9, scored significantly higher than unfermented and monoculture fermented juice. Among these, S10, S14, and S7 scored highest in the floral aroma profile. This indicates that the aromatic complexity of fermented mulberry juice is intricately composed of both floral and fruity aromas [4]. In the mellow aroma profile, C, S0, and S5 scored much higher than other samples but all samples scored the lowest with thin and weak aroma. The contribution to estery and delicate aroma profile to the samples was very weak and small and there is no significant difference among samples. Ultrasonication and fermentation improved the flavor and taste of the mulberry juice [27]. The sensory evaluation findings concerning aroma attributes exhibited heightened quality, characterized by vigorous aromatic profiles and a well-balanced, harmonious product. The ultrasound assisted fermentation (mono and coculture) also imparts much better and enriched aroma compounds in mulberry juice.

### 3.7 Molecular docking modeling and correlation analysis

**Fig. 6:**
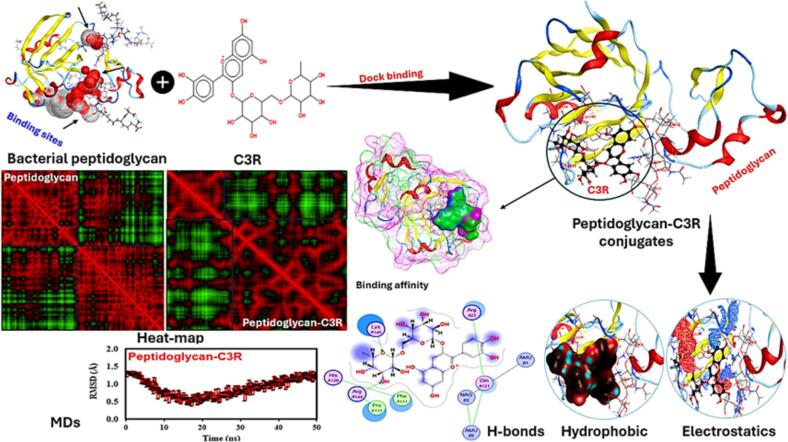
Peptidoglycan-C3R interaction scheme of the bacterial cell wall and anthocyanin structures with representing their binding affinity, overall conjugates, hydrophobicity alterations, electrostatic potentials, H-bonding, heat-map analysis, and MDs-analysis. The end conjugates showed a binding score and energy affinity of −1.02 and −1.83 kcal/mol, separately.

**Fig. 7:**
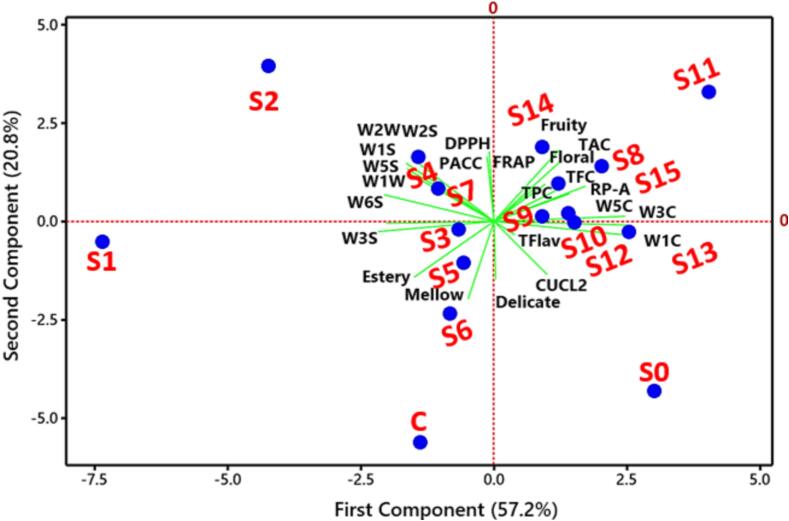
PCA analysis of the correlation between all treatments and the antioxidative polyphenols, sensorial parameters, and E-nose based parameters.

Fig. 6 presents a detailed visualization of the peptidoglycan-C3R interaction including a variety of analyses to clarify the molecular dynamics and binding affinities associated with this interaction and highlighting their binding sites. Peptidoglycans interact with C3R *via* different forces, indicating a strong binding potential that enables the formation of peptidoglycan-C3R conjugates. The analysis of binding affinity is essential, as it offers valuable insights into the strength and stability of protein–ligand interaction. The binding score of −1.02 and an energy affinity of −1.83 kcal/mol suggest a stable interaction, indicating a moderate binding strength that may impact the functional properties of the conjugates. There are notable differences in the binding interactions when comparing the heat map of peptidoglycan alone to the peptidoglycan-C3R conjugates. The modified patterns in the heat map indicate that C3R was well interacted with peptidoglycan, where this interaction might be responsible for maintaining the stability of C3R and other polyphenols (agreeing well with our experimental results). The analysis conducted by the MDs provides additional support, as it demonstrates the RMSD over time. The RMSD plot provides insights into the structural stability and conformational fluctuations of the peptidoglycan-C3R conjugates throughout MD-simulations. The consistent RMSD values indicate that the conjugates retain their structural integrity over time. Elaborate diagrams highlight precise interactions within peptidoglycan-C3R conjugates. H-bonding is essential for maintaining the stability of protein structures. The diagram illustrates the amino acids that play a crucial role in these interactions, demonstrating the impact of protein binding on their overall conformation and stability. For example, Lys140 and Arg144 of peptidoglycan directly interacted with the anthocyanin structure. Hydrophobic interactions play a crucial role in maintaining the structural integrity of proteins. The visualization demonstrates the alterations in hydrophobic regions, indicating the impact of the conjugation with C3R on the hydrophobic core of peptidoglycan. Electrostatic interactions play a crucial role in determining the strength and selectivity of protein–protein interactions, where other 8 amino acids of peptidoglycan interacted with C3R. The visualization of electrostatic potentials demonstrates the changes in charge distribution that occur during binding, impacting the strength of binding and functional properties of the conjugates. PC_1_ positively correlated with all the juice parameters including some of the E-nose, sensorial, and bioactive components *i.e.,* FRAP, TPC, TFC, RP-A, TFlav, CUCL2, Fruity, Floral, Delicate, W5C, W3C, and W1C (Fig. 7). Meanwhile, PC_1_ positively correlated with S0 and S8-15. PC_2_ positively correlated with S2, S4, S7, S8, S9, S10, S14, and S15, showing their closed parameters, where most of the other parameters were also correlated with this component analysis.

## 4 Conclusion

The study's findings highlight the significant potential of multi-frequency ultrasound-assisted fermentation in enhancing the quality of mulberry juice. This innovative approach not only improves antioxidant capacity and phenolic content but also optimizes sensory attributes, making the juice more appealing to consumers. The observation of a more porous microstructure and increased bioactive compounds, particularly in co-culture fermented samples, underscores the effectiveness of this method in producing high-quality functional beverages. To build on these promising results, future research should focus on optimizing fermentation conditions by investigating various parameters such as temperature, duration, and frequency of ultrasound treatment to achieve even higher yields of bioactive compounds and improved sensory qualities. Additionally, exploring diverse probiotic strains of LAB may further enhance the functional properties of mulberry juice, offering a broader spectrum of health benefits. Assessing the long-term stability of bioactive compounds will be crucial for determining the shelf-life and overall viability of ultrasound-assisted fermented mulberry juice in commercial applications. Understanding consumer preferences and acceptance will also be essential for a successful market introduction. Finally, extending this methodology to other fruit juices could provide insights into its versatility and effectiveness across different substrates. By addressing these aspects, future research can significantly contribute to the development of functional beverages that not only meet consumer demands but also promote health benefits through enhanced nutritional profiles.

## Ethics statement

No animal and human experiments. The sensory study was performed in a safe and hygienic food production environment, with the generous assistance of the School of Food and Biological Engineering at Jiangsu University, China.

## CRediT authorship contribution statement

**Sanabil Yaqoob:** Writing – original draft, Methodology, Investigation, Formal analysis, Conceptualization. **Aysha Imtiaz:** Writing – original draft, Validation, Methodology, Data curation, Conceptualization. **Ibrahim Khalifa:** Writing – review & editing, Writing – original draft, Visualization, Validation, Conceptualization. **Sajid Maqsood:** Writing – review & editing, Validation, Software, Project administration, Formal analysis. **Riaz Ullah:** Writing – review & editing, Writing – original draft, Visualization, Validation. **Abdelaaty A. Shahat:** Writing – review & editing, Writing – original draft, Validation, Software, Resources. **Fahad Al-Asmari:** Writing – review & editing, Writing – original draft, Visualization, Software, Resources, Project administration. **Mian Shamas Murtaza:** Writing – original draft, Visualization, Software, Methodology. **Jian-Ya Qian:** Writing – review & editing, Writing – original draft, Visualization, Validation, Supervision, Resources. **Yongkun Ma:** Writing – review & editing, Writing – original draft, Visualization, Supervision, Resources, Project administration.

## Declaration of competing interest

The authors declare that they have no known competing financial interests or personal relationships that could have appeared to influence the work reported in this paper.
