# Supplementary material for: Multi-frequency sono-fermentation with mono and co-cultures of LAB synergistically enhance mulberry juice: Evidence from metabolic, micromorphological, sensorial, and computational approaches
Source: Ultrason Sonochem. 2024 Oct 22;111:107117. doi: 10.1016/j.ultsonch.2024.107117 (PMC11541811; doi:10.1016/j.ultsonch.2024.107117)
Supplement: Supplementary Data 1 [file mmc1.docx]

**Table 1.** Treatment codes for samples.

| **No.** | **Codes** | **Treatment** | **Description** |
| --- | --- | --- | --- |
| 1 | C | C | Mulberry juice control. |
| 2 | S_o_ | US-C | Multi-frequency ultrasonicated treated juice. |
| 3 | S_1_ | US-LC | Multi-frequency ultrasonicated assisted fermentation with *Lactobacillus casei.* |
| 4 | S_2_ | US-LP | Multi-frequency ultrasonicated assisted fermentation with *Lactobacillus plantarum.* |
| 5 | S_3_ | US-LPC | Multi-frequency ultrasonicated assisted fermentation with *Lactobacillus paracasei.* |
| 6 | S_4_ | US-LA | Multi-frequency ultrasonicated assisted fermentation with *Lactobacillus acidophilus.* |
| 7 | S_5_ | US-LH | Multi-frequency Ultrasonicated assisted fermentation with *Lactobacillus helveticus.* |
| 8 | S_6_ | US-LC-LP | Multi-frequency ultrasonicated assisted fermentation with *Lactobacillus casei- Lactobacillus plantarum.* |
| 9 | S_7_ | US-LC-LPC | Multi-frequency ultrasonicated assisted fermentation with *Lactobacillus casei- Lactobacillus paracasei.* |
| 10 | S_8_ | US-LC-LA | Multi-frequency ultrasonicated assisted fermentation with *Lactobacillus casei- Lactobacillus acidophilus.* |
| 11 | S_9_ | US-LC-LH | Multi-frequency ultrasonicated assisted fermentation with *Lactobacillus casei- Lactobacillus helveticus.* |
| 12 | S_10_ | US-LP-LPC | Multi-frequency ultrasonicated assisted fermentation with *Lactobacillus plantarum- Lactobacillus paracasei.* |
| 13 | S_11_ | US-LP-LA | Multi-frequency ultrasonicated assisted fermentation with *Lactobacillus plantarum- Lactobacillus acidophilus.* |
| 14 | S_12_ | US-LP-LH | Multi-frequency ultrasonicated assisted fermentation with *Lactobacillus plantarum- Lactobacillus helveticus.* |
| 15 | S_13_ | US-LPC-LA | Multi-frequency ultrasonicated assisted fermentation with *Lactobacillus paracasei- Lactobacillus acidophilus.* |
| 16 | S_14_ | US-LPC-LH | Multi-frequency ultrasonicated assisted fermentation with *Lactobacillus paracasei- Lactobacillus helveticus.* |
| 17 | S_15_ | US-LA-LH | Multi-frequency ultrasonicated assisted fermentation with *Lactobacillus acidophilus- Lactobacillus helveticus.* |
